# Rectal Sparing Approaches after Neoadjuvant Treatment for Rectal Cancer: A Systematic Review and Meta-Analysis Comparing Local Excision and Watch and Wait

**DOI:** 10.3390/cancers15020465

**Published:** 2023-01-11

**Authors:** Quoc Riccardo Bao, Stefania Ferrari, Giulia Capelli, Cesare Ruffolo, Marco Scarpa, Amedea Agnes, Giuditta Chiloiro, Elisa Palazzari, Emanuele Damiano Luca Urso, Salvatore Pucciarelli, Gaya Spolverato

**Affiliations:** 1 General Surgery 3, Department of Surgical, Oncological and Gastroenterological Sciences (DiSCOG), University of Padova, 35128 Padova, Italy; 2 Department of Surgery, ASST Bergamo Est, 24068 Bergamo, Italy; 3 Department of Surgery, Fondazione Policlinico Universitario A. Gemelli—IRCCS, 00168 Roma, Italy; 4 Department of Radiation Oncology, Fondazione Policlinico Universitario A. Gemelli—IRCCS, 00168 Roma, Italy; 5 Department of Radiation Oncology, Centro di Riferimento Oncologico di Aviano (CRO)—IRCCS, 33081 Aviano, Italy

**Keywords:** rectal sparing approach, watch and wait, wait and see, local excision

## Abstract

**Simple Summary:**

The increasing rate of complete response following neoadjuvant treatment for locally advanced rectal cancer has allowed an increasing interest for rectal-sparing approaches. Local excision and Watch and Wait allow the ability to avoid all the consequences and sequelae of Total Mesorectal Excision. However, despite excellent results in term of quality of life and bowel function, some concerns have been raised regarding the long-term outcomes. Most of the current studies compared Local Excision or Watch and Wait with Total Mesorectal excision, but only very few studies compare these two approaches.

**Abstract:**

Local Excision (LE) or Watch and Wait (WW) for patients with complete clinical response or near-complete clinical response after neoadjuvant chemoradiotherapy (nCRT) were proposed to avoid morbidity and impairment of quality of life after rectal resection. The aim of this study is to perform a systematic review of the literature, and to compare rectal-sparing approaches, in terms of rectum-preservation rate, local control, and distant recurrences. A systematic review and meta-analysis were performed of studies published until July 2022 (PROSPERO, registration CRD42022341480), and the quality of evidence was assessed using a GRADE approach. Seven retrospective studies and one prospective trial were included. In six studies, patients were treated with standard long-course nCRT, and in two with Total Neoadjuvant Therapy (TNT). Overall, there were 213 and 188 patients in WW and LE group, respectively, and no difference was found between WW and LE when considering rectum-preservation rate (OR 0.80 95%CI 0.31–2.01, *p* = 0.63), local disease (OR 1.60 95%CI 0.75–3.42, *p* = 0.22), locoregional failure (OR 0.85 95%CI 0.20–3.66, *p* = 0.83) and distant recurrence (OR 0.76 95%CI 0.37–1.55, *p* = 0.45). Studies directly comparing WW and LE are still lacking, even though no differences between WW and LE in terms of rectum-preservation, local control, and distant recurrences have been found.

## 1. Introduction

The standard care for locally advanced rectal cancer is currently neoadjuvant chemoradiotherapy (nCRT) followed by total mesorectal excision (TME) [1]. This approach has been proven to have excellent results in terms of survival and recurrence, but it is associated with several complications and impairment of the patient’s quality of life [2].

According to recent studies, up to 30% of patients show complete response to nCRT [3]. Rectal-sparing approaches, such as Local Excision (LE) or Watch and Wait (WW), were proposed to avoid surgical consequences, for the management of selected patients that show a complete clinical response (cCR) or near complete response (nCR) after neoadjuvant treatment. WW policy consists of a strict follow-up after a cCR, allowing a surgery-free treatment, which would be considered lately only in case of tumor local regrowth. On the other hand, LE consists in a full-thickness excisional macro-biopsy of the residual tumor/tumor scar, and was also proposed in case of cCR or nCR. This approach can be performed also using minimally invasive transanal approaches, such as transanal endoscopic microsurgery (TEM), transanal minimally invasive surgery (TAMIS) and transanal endoscopic operation (TEO), and offers the advantage of a histopathological proof of cCR or nCR, and to avoid a rectal resection and its post-operative complications and morbidity [4,5].

In recent years there has been increasing interest in these rectal-sparing strategies. Nevertheless, only few studies directly compared the two different approaches, in particular in terms of rectum-preservation, recurrence and survival. Most recently, the WW approach has been proven to have excellent outcomes, including when surgery is needed to treat tumor regrowth [6]. Most recently, rectum-preservation using a WW policy was reached in half of the patients with a locally advanced rectal cancer following total neoadjuvant therapy (TNT), with no difference in term of DFS in patients undergoing TME for incomplete response and TME for tumor local regrowth [7]. Promising long-term outcomes were also reported following LE, with a 5- and 10-year OS of 87 and 79%, respectively [5]. To note, LE permits to candidate patients a completion of TME surgery, in order to avoid recurrence in cases of a non-radical, persistent or aggressive tumor, or in case of misdiagnosis and clinical and pathological discrepancy.

In the present literature, data regarding the outcomes of rectal-sparing approaches are available, but only few studies reported and directly compared WW and LE. The aim of this study is to perform a systematic review of the current evidence of study comparing rectal-sparing strategies, and to perform a meta-analysis of the rate of rectum-preservation rate, local control and distant recurrences (DR).

## 2. Methods

### 2.1. Study Registration

The study protocol has been registered on the International Prospective Register of Systematic Reviews (PROSPERO, registration number CRD42022341480) on 8 July 2022.

### 2.2. Literature Search and Review, and Studies Selection

A systematic review of the published literature until 1 July 2022 was performed in the following databases: PubMed, Embase, Scopus, Web of Science. The keywords used included “rectal cancer,” “organ sparing,” “rectum sparing,” “local excision,” “watch and wait,” “wait and see,” “conservative approach,” and ”non operative management,” in combination with Boolean operators (AND, OR, NOT). Two reviewers (Q.R.B. and S.F.) independently reviewed titles and abstracts and cross-checked the results of the studies. Disagreements were settled by a third reviewer (M.S.). Original articles that fulfilled the inclusion criteria were recruited for full-text evaluation. The systematic review was performed according to Preferred Reporting Items for Systematic Review and Meta-Analyses (PRISMA) guidelines [8].

### 2.3. Inclusion and Exclusion Criteria

All studies reporting clinical outcomes of WW and LE after a cCR or nCR were considered. Randomized controlled trials, controlled clinical trials, observational, cross-sectional, retrospective and prospective studies were considered if the data were reported as follows: rectum-preservation rate or number of patients that underwent TME or rectal resection; local regrowth after WW and local recurrence (LR) after LE; DR after both WW and LE. Only articles in English were considered. Studies were included if they contained data from patients with rectal cancer after nCRT with cCR or nCR and if they reported separately outcomes of LE and WW. Studies also reporting a comparison of TME were considered for inclusion, if outcomes of WW and LE were reported separately.

Editorials, review articles, invited commentaries and case reports were excluded. Studies reporting WW or LE for incomplete clinical response, or LE without nCRT were also excluded. Studies not reporting separately the outcomes of WW and LE were not included. In case of studies with overlapping cohort, the most recent study was included.

### 2.4. Data Extraction and Statistical Methods

The primary outcome was the rectum-preservation rate. In case of study not reporting the overall rectum-preservation rate, in the study reporting the outcomes after WW and LE, rectal resection/TME, both for local regrowth or LR, or for any causes including complications, were considered as event. Re-LE was considered a rectal-sparing procedure. Secondary outcomes of the study were local control of the disease, and distant recurrence. Regarding local control, we decided to compare the presence of local disease, and the events considered were local regrowth in WW group, and LR in LE group, even if technically their definition are different, but both potentially impacting on the risk of a rectal resection and a local or distant recurrences. Locoregional failure was also part of local control analysis, and was defined as not respectable or not-treated local regrowth or LR. DR was defined as any recurrence outside the pelvis. A meta-analysis comparing these outcomes in patients followed in a WW program versus treated with LE was performed using Review Manager ([*Computer program*]. *Version 5.4*, Cochrane Collaboration, 2020). A random effects model was applied to obtain a pooled odds ratio (OR) and 95% confidence interval (95%CI). The heterogeneity of the studies was assessed using the I2 statistic. A statistical significance was set for *p* < 0.05.

### 2.5. Quality Evaluation of Evidence

A GRADE approach was used to evaluate the quality of evidence from included studies, starting at high quality and downgrading for risk of bias, imprecision, inconsistency, indirectness and publication bias [9] by using GRADEproGDT (https://gdt.gradepro.org/, accessed on 31 October 2022).

## 3. Results

### 3.1. Study Selection

**Figure 1:**
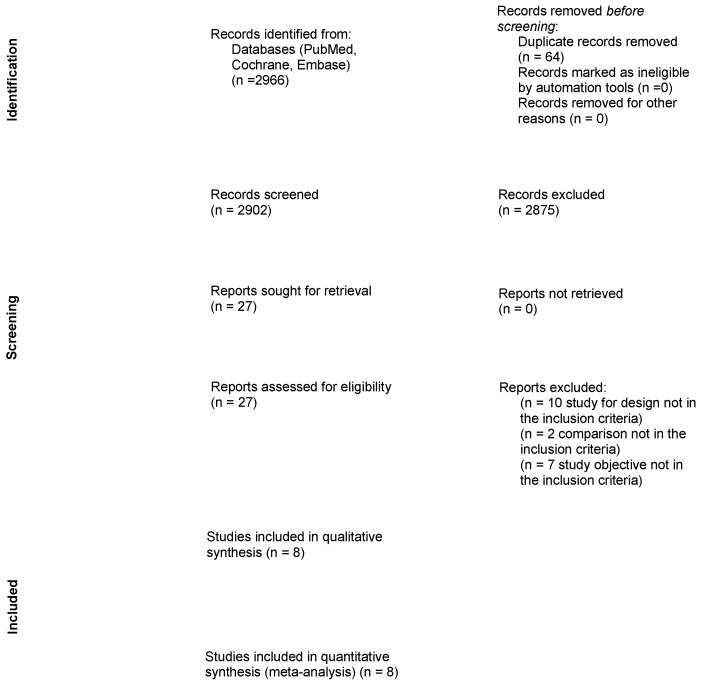
Prisma-flow chart.

Overall, 2966 articles were screened and 27 were identified to undergo full-text review. After the removal of unavailable and irrelevant articles, eight studies were selected for the systematic review and meta-analysis (Figure 1). Among the eight included studies published between 2016 and 2022, six were retrospective single-center studies, one was a multicenter retrospective study, and one was a single-center phase II clinical trial.

### 3.2. Study Design, Definition of Clinical Response, and Aspects of Staging and Treatment

Study design, nCRT type, assessment and definition of clinical response were summarized in Table 1. Out of eight included studies, six were retrospective studies including patients treated with a standard long-course CRT. Al-Najami et al. reported patients treated with WW or LE between 2017 and 2018 at Oxford University after standard nCRT, consisting of 45–50.8 Gy radiotherapy with concomitant Capecitabine/5FU chemotherapy, since 2015 an intensity-modulated radiotherapy. The term cCR was defined, as previously defined by Sammour et al. 4 months since the completion of nCRT [10]. No patients had consolidation or adjuvant therapy [11]. Park et al. retrospectively reported oncologic outcomes of 42 patients treated with LE, and 32 with WW. Rectal-sparing strategies were proposed to patients with cCR or nCR, even if cCR and nCR were not defined, and the indication for WW or LE were limited to surgeon-patients discussion. Completion-TME after LE was recommended in patients with ypT ≥ 2, or in patients with ypT1, SM ≥ 2, lymphovascular invasion, perineural invasion, tumor budding or margin involvement. Vaccaro et al. reported 30 patients with cCR and nCR treated between 2005 and 2014. The term cCR was defined as described by Habr Gama et al. [12], whereas nCR was defined by the clinical or radiological persistence of a mobile, non-ulcerated lesion smaller than 2 cm, and were considered for surgery (TME or LE). The decision for TME or LE took into account distance to anal verge and patient/surgeon preference [13]. Creavin et al. reported 60 patients treated with rectal-sparing approaches collected in their prospective database. Patients with a cCR were offered a WW program, while patients with residual ulcer were treated with TAE, TEM or TAMIS. Completion TME was offered to patients with ypT ≥ 2 or close excision margin [14]. Yeom et al. retrospectively collected the patients with a cCR, defined at radiological restaging as no residual tumor or tumor fibrosis and no suspicious metastatic lymphnodes at MRI. All patients were offered a standard TME, but WW or LE were considered whenever sphincter-saving surgery was not possible or patients’ refusal [15]. Lee et al. reported a retrospective cross-sectional analysis in three Korean centers. A rectal-sparing approach was offered to patients with a cCR 4 to 12 weeks after the end of nCRT. The LE approach was at surgeon discretion, and required a full-thickness excision of the mesorectum with adequate tumor margin and no tumor on frozen section [16].

Two included studies reported the outcomes after TNT. Asoglu et al. retrospectively reported 66 patients treated with 6 weeks of CRT (50.4 Gy in 28 fractions with concomitant capecitabine) followed by a restaging at 4 weeks, and in case of a clinical response >50%, the patients were treated with consolidation chemotherapy (6 cycles of FOLFOX/CAPEOX). Four weeks from the end of consolidation treatment, the patients were re-staged with sigmoidoscopy, MRI and PET-CT. Decision on the following treatment was assessed at a multidisciplinary team after restaging, even if no details regarding the indication for LE instead of WW are specified [17]. Wang et al. reported a phase II trial which included patients with a locally advanced rectal cancer treated with TNT. All patients were treated with intensity-modulated radiation therapy, consisting of 50.6 Gy in 22 fractions with concurrent Capecitabine, followed by 4 cycles of consolidation CAPEOX. Final evaluation of tumor response was taken 16 to 20 weeks after completion of TNT. cCR and nCR were defined according the MSKCC criteria [18]. After multidisciplinary team discussion, patients with cCR were proposed to receive a WW, whereas patients with a nCR were recommended to undergo WW or LE (TAE or TAMIS). The primary endpoint was the 3-year rectum-preservation rate, defined as percentage of patients with a cCR or nCR treated with a rectal-sparing approach [19].

**Table 1:** Design of the study, type of neoadjuvant treatment, assessment and definition of Complete Clinical Response (cCR).

Author	Year	Design of the Study	NAT	Assessment of Clinical Response		Definition of Complete Clinical Response	Indication for Completion Surgery Following LE
				Timing (Weeks)	Exams		
Vaccaro	2016	Retrospective, monocenter	LCCRT	8–12	Rectoscopy, CEA, MRI, and CT scan.	Flat mucosa or white scar, teleangectasia.	Not reported
Creavin	2016	Retrospective, monocenter	LCCRT	6–8	DRE, sigmoidoscopy, CEA, MRI, and CT scan.	Visible scar only or residual scar/ulcer <3 cm	ypT ≥ 2 Positive margins
Asoglu	2019	Retrospective, monocenter	TNT	4	Sigmoidoscopy, MRI, PET-CT	Flat mucosa or white scar, teleangectasia. MRI and PET-CT complete response, MRI TRG 1–2, absence of FDG uptake	Not reported
Park	2019	Retrospective, monocenter	LCCRT	4–6	DRE, sigmoidoscopy, CT, MRI	Not defined	ypT ≥ 2 ypT1, with SM ≥ 2, LNI, PNI, tumor budding, positive margins
Yeom	2019	Retrospective, monocenter	LCCRT	5–6	MRI	No residual tumor or residual fibrosis; no suspicious metastatic lymph nodes	Not reported
Al-Najami	2021	Retrospective, monocenter	LCRRT	16	DRE, sigmoidoscopy, MRI	Flat mucosa or white scar, MRI TRG1	Not reported
Lee	2021	Retrospective, multicenter	LCCRT	4–12	DRE, sigmoidoscopy, CEA, MRI, and CT scan.	Scar or small ulcer, ycT0N0	Not reported
Wang	2022	Phase II, monocenter	TNT	16–20	DRE, sigmoidoscopy, CEA, MRI, and CT scan.	Flat, white scar, no ulcer, no nodularity. DRE: normal. MRI: T2 dark signal, no visible lymphnodes, DWI no visible signal, lack/low signal ADC	Poor differentiation, LNI, PNI, positive margins

Abbreviations: NAT neoadjuvant treatment; LCCRT long-course chemoradiotherapy; TNT total neoadjuvant therapy; CEA Carcinoembryonic antigen; MRI magnetic resonance imaging; CT computed tomography, DRE digital rectal examination; PET positron emission tomography; TRG tumor regression grade; FDG fluorodeoxyglucose; DWI: diffusion weighted imaging; ADC apparent diffusion coefficient; LNI lymphovascular invasion; PNI perineural invasion.

### 3.3. Post-Treatment Complications and Short-Terms Outcomes

Only two studies provided details regarding the post-LE complications. Park et al. reported 23% of post-operative complications that occurred in LE group. The most common complication was anal pain, occurring in six (14.3%) patients. Other complications included incontinence (reported in two patients), voiding difficulty (reported in one patient), and perirectal inflammation in (reported in one patient). One patient required a stoma for a pelvic abscess. Post-operative complications of salvage TME surgery for local regrowth WW group were also described. Out of nine patients, three (33.3%) developed post-salvage surgery complications in WW group. Two patients had ileus and voiding difficulty, while one patient required surgical resection for internal herniation [20]. Lee et al. also described post-operative complications in LE group, which did not differ from TME group (13.3% vs. 22.6%, *p* = 0.348). One patient required a temporary ileostomy that was performed for LE dehiscence, one (3.3%) patient had ileus and two (6.7%) patients had intraluminal bleeding. Furthermore, one patient underwent APR after 2 months for severe anal incontinence [16]. No data were available for post-salvage-surgery complications in the WW group.

Asoglu et al. mentioned that three of six patients that underwent LE had a wound detachment [17]. Six patients were excluded from the analysis due to disease progression during TNT (four patients), myocardial infarction (one patient) and liver toxicity (one patients). In the study by Wang et al., the toxicity due to TNT was also reported. Overall, 87% of enrolled patients completed the consolidation chemotherapy, while eight patients had a cycle or dose reduction, and two patients did not receive consolidation chemotherapy. Overall, grade three to four toxicities occurred in one third of the patients [19].

Stoma-free rate and stoma-free survival in patients followed in a WW program or following a LE were not reported by any study.

### 3.4. Local Regrowth, Local and Distant Recurrences, Rectum-Preservation Rate

Rectum-preservation rate, local regrowth, LR, and DR are summarized in Table 2. Al Najami et al. collected data on 42 patients in the WW group following a cCR; six (14.3%) of them had a local regrowth within 1 year, and 11 (26.2%) within 2 years. Only two patients underwent salvage surgery. The LE group included 22 patients, and none of them had a pCR at final histopathologic report, and only six (27%) had a ypT1. Most of the patients (n = 16, 72.7%) had a ypT2, even if only three patients had a completion surgery (refusal n = 7, unfit for surgery n = 6). Overall, 10 (45.5%) patients had a LR, and only two underwent salvage surgery. The rates of rectum-preservation were 95.2% and 73.0% in WW and LE patients, and the 3-year DFS was 74.9 vs. 66.2% in WW and LE patients, respectively [11]. Park et al. included 42 patients in the LE group; a total of 22 were confirmed to have a pCR, whereas 14 had a ypT1 and five had a ypT ≥ 2. To note, overall 11 patients were recommended for completion TME, but none of them agreed to undergo surgery. Among them, five experienced recurrences (LR = 1, DR = 3, LR + DR = 1), and all of the patients had their rectum spared at the last follow up. In the WW group (n = 32), 10 patients had a local regrowth, five of these was early local regrowth (within one year), one patient with concomitant DR. Six of them underwent salvage TME surgery, and five of them experienced recurrence after salvage surgery. The overall rectal sparing rate was 94.6%, which was significantly higher in LE compared to the WW group (100% vs. 87.5%, *p* = 0.02). The 5-year RFS was 84.6 and 69.8% *p* = 0.12) in LE and WW group, respectively [20]. In the study by Yeom et al., 15 patients were followed in a WW program, and 25 were treated with LE. Following LE, a pCR were reported in 12 (48.0%) patients, a ypT1–2 in 11(44.0%), and ypT3–4 in 2(8.0%). Indications and data regarding completion TME were not reported. At a median follow-up of 30 (2–93) months, 4 (16.0%) patients had a LR, 2(8.0%) underwent salvage TME, 1 re-LE. Overall, four (16.0%) patients had LR + DR, and one (4.0%) patient a DR only. In the WW group, three (20.0%) patients had a local regrowth, two (13.3%) patients a DR, and one (6.7%) patient LR + DR. Only one patient underwent a salvage TME, one was treated with chemotherapy, while the remaining refused any treatment. The 3- and 5-year LRFS were 76.8 and 53.8%, and 78.6 and 26.9% in the LE and WW groups, respectively. The 3- and 5-year DFS were 72.9 and 55.5%, and 72.9 and 27.8%, respectively. At regression analysis, a WW approach was associated with a worse LRFS [15].

Vaccaro et al. included 23 patients with a cCR and followed in a WW program, and seven with a nCR treated with a LE. In the WW group, four (17.4%) patients had a local regrowth, and two of them had salvage surgery. The rectum-preservation rate was 91%, and 3-year DFS was 94.1%. In the LE group, most of the patients had a ypT2 at final histopathological exam. None of them had LR, even if one had liver and lung metastases after 8 and 15 months requiring surgical treatment. None of the patients treated with LE had a TME or a rectal resection [13]. In the study by Creavin et al., 10 patients had a WW and 50 a LE. Out of 50 patients treated with LE, 26(52.0%) had a pCR, whereas completion-TME was performed in 15(30%) patients for ypT2 ≥ 2 or close (<1 cm) resection margins. At a median follow-up of 29 (12–49) months, in LE group OS and DFS were 94.3 and 80% respectively, accounting for five (10.0%) patients with DR, and three (6.0%) patients with LR. Patients in WW group, at a median follow-up of 42 (22.5–55.2) months, reported also a DFS of 80%, accounting for one (10.0%) patient with a local tumor regrowth treated with TME, and one (10.0%) with a DR who deceased for disease progression. The overall rates of rectum-preservation were 66% and 90% in the LE and WW group, respectively [14]. Lee et al. included 30 patients treated with LE and 14 followed in WW program. One patient had an APR for anal incontinence following LE. Among four (13.3%) patients with LR (ypT0 = 1, ypT1 = 3), one had an re-LE, two had a salvage TME, and one refused any treatment. Among three (21.4%) patients who experienced a local regrowth, two required a TME surgery, and one refused any treatment. At survival analysis, OS and DFS did not differ in LE and WW, and the rate of rectum-preservation was 85.7% and 90% in WW and LE, respectively [16].

In the study by Asoglu et al., out of 39 patients included in the WW group, six (15.4%) had local regrowth, three had a salvage TME, and three a LE. Two (5.1%) patients experienced a DR, both within 2 years. In the LE group (n = 6), one (16.7%) patient had a DR, whereas LR occurred in one (16.7%) patient requiring a salvage TME [17]. In the phase II study by Wang et al., 33 (51.6%) had a cCR and 13 (20.3%) nCR following TNT. According to the study protocol, 38 patients were enrolled in WW group, six patients in the LE group, and the remaining 12 patients underwent TME. None of the patients in the LE group required a completion TME. In the WW group, at a median follow-up of 31.6 months, 7 (18.4%) patients experienced a local regrowth, all within the first year. The estimated 2-year regrowth-free rate was 12.9% and 42.9% in cCR and nCR patients, respectively. Six patients underwent salvage surgery, including two LE and four TME. No LR were observed in LE group. DR occurred in one (2.6%) patient in WW and in 1 (16.7%) patient treated with LE. The 3-year rectum-preservation rate of the entire cohort was 67.2%, with a 3-year stoma-free survival of 82.7%. The 3-year cancer-specific survival and non-regrowth DFS were 96.6 and 92.2%, respectively [19]. 

**Table 2:** Number of patients, and long-term outcomes.

Author	Year	Patients		Completion TME in LE	Follow-Up		Local Regrowth	Local Recurrence	Distant Recurrence		Organ Preservation		Disease-Free Survival (DFS)	
		WW	LE	Required/Performed	WW	LE			WW	LE	WW	LE	WW	LE
Vaccaro	2016	23	7	0/0	46		4 (18.5)	0 (0)	1 (4.3)	1 (14.3)	21 (91)	7 (100)	3-year 94.1%	
Creavin	2016	10	50	15/15	42	29	1 (10)	3 (6)	1 (10)	5 (10)	9 (90)	33 (66)	80%	80%
Asoglu	2019	39	6	0/0	22		6 (15.4)	1 (16.6)	2 (5.1)	1 (16.6)	36 (92.3)	5 (83.3)		
Park	2019	32	42	11/0	73	65	10 (31.3)	2 (4.8)	1 (3.1)	4 (9.5)	27 (84.3)	42 (100)	5-year RFS 69.8%	5-year RFS 84.6%
Yeom	2019	15	25	NR	20	30	4 (26.7)	4 (16)	5 (33.3)	6 (24)	14 (93.3)	23 (92.0)	3-year DFS 72.9%	3-year DFS 55.5%
Al-Najami	2021	42	22	16/3		53	11 (26)	10 (45)		4 (18.2)	38 (95)	16 (73)		3-year 66.2%
Lee	2021	14	30		64.7		3 (21.4)	4 (13.3)	0 (0)	2 (6.7)	12 (85.7)	27 (90)	5-year DFS 76.4%	
Wang	2022	38	6	0/0	39.5		7 (18.4)	0 (0)	2 (5.3)	1 (16.7)	33 (86.8)	6 (100)		

### 3.5. Watch and Wait vs. Local Excision: Long-Term Outcomes Meta-Analysis

**Figure 2:**
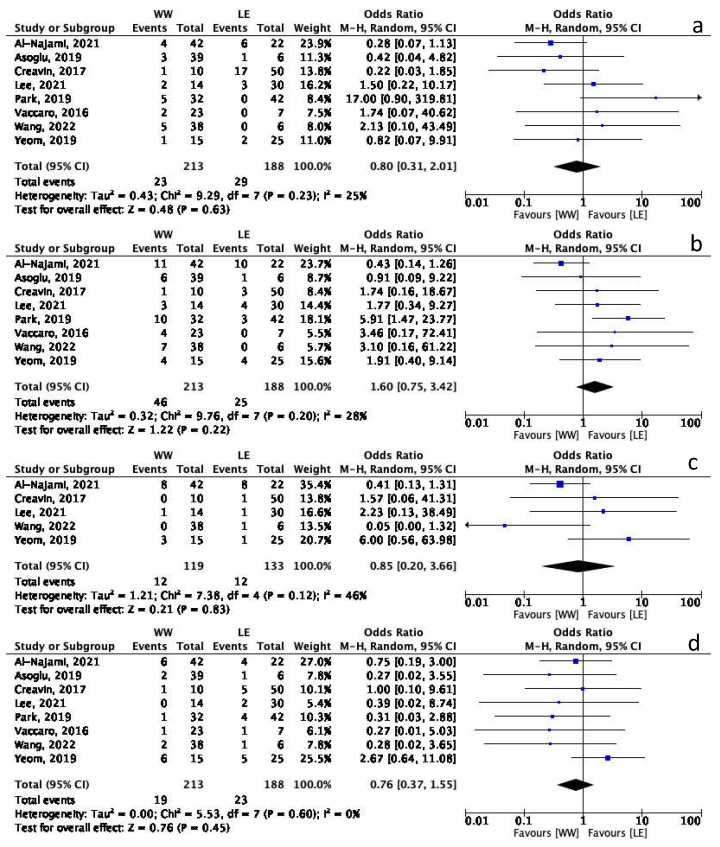
Meta-analysis Forest plot. (**a**) Rectum-preservation. (**b**) Local disease. (**c**) Locoregional failure. (**d**) Distant recurrence [11,13,14,15,16,17,19,20].

A meta-analysis comparing WW and LE was performed including the eight above mentioned studies regarding rectum-preservation rate, local disease and distant recurrence. Overall, 231 patients were included in the WW group while 188 were included in LE. There was no difference between WW and LE when considering rectum-preservation rate (OR 0.80 95%CI 0.31–2.01, *p* = 0.63) with a rate of rectum-preservation of 89.2 vs. 84.6% in WW and LE, respectively. No differences were also found when comparing local disease (21.6% vs. 13.3% in WW and LE, respectively) (OR 1.60 95%CI 0.75–3.42, *p* = 0.22), and DR (8.9% vs. 12.2% in WW and LE, respectively) (OR 0.76 95%CI 0.37–1.55, *p* = 0.45) (Figure 2).

Data regarding locoregional failure were available in only five studies, including 119 patients in WW group and 133 in LE group. No difference in terms of locoregional failure was found between the groups (10.0% Vs 9.0% in WW and LE, respectively, OR 0.85 95%CI 0.20–3.66, *p* = 0.83) (Figure 2c). The heterogeneity of these studies was low.

### 3.6. Overall Quality of Evidence

According to the GRADE approach (Supplementary Table S1), the overall quality of evidence was very low for all the outcomes analyzed. First, only one phase-2 trial was included, whereas the other seven studies were retrospective. The risk of bias for the included studies was considered very serious, mainly due to the absence of control of confounding factors, and to the short follow-up of most of the studies. Only three studies had a median follow-up longer than 5 years. Overall, as a confounding factor, most of the studies included patients with a cCR or nCR without specific recommendations for WW or LE. Furthermore, most of the studies did not include a definition for completion surgery following LE. The rate of inconsistency was considered serious for the lack of description of study methods. The rate of indirectness of evidence was also considered serious, considering that only Wang et al. had the rectum-preservation rate as primary endpoint [19], whereas the other studies mainly focused on survival mainly. The imprecision dimension was judged to be serious for rectum-preservation, locoregional failure and distant recurrences, accounting for the low number of events.

## 4. Discussion

The aim of the current study was to systematically review the evidence of available literature regarding rectal-sparing approaches, and to compare the outcomes of WW and LE after nCRT. In our meta-analysis we included eight studies, and we found no differences in terms of rectum-preservation, local disease, locoregional failure and DR. Most of the included studies were retrospective, while one was multicenter, and one was prospective.

Currently, there are very few data available comparing WW and LE in rectal cancer following nCRT, while studies focused on comparing rectal-sparing approaches with TME are more often reported. These protocols are still used in clinical settings only, showing good results in terms of rectum-preservation. In the present meta-analysis, no differences in terms of rectum-preservation were found. In the included studies, the rate of rectum-preservation following LE ranged between 66 and 100%, and it is mainly affected by the number of patients who underwent completion TME following LE when negative histopathological features are present. In the prospective trial, after nCRT + LE more than 30% of the patients required a completion surgery [5,21]. Indications for completion surgery may also be slightly different according to the study protocol, as in the GRECCAR 2 trial which recommended completion for ypT ≥ 2 or positive margins [21], whereas other studies also included ypT1 with high-risk features [4,5]. As reported in Table 1, most of the included studies (5 of 8) lacked indication for completion surgery, and completion TME was generally recommended in ypT ≥ 2 or positive margins (R1), or in ypT1 with SM ≥ 2, lympho-vascular invasion, perineural invasion, tumor budding, poor differentiation [14,19,20]. However, the rate of refusal or unperformed completion surgery was extremely high in all the included studies, such as in the study by Park et al., where all of the 11 patients recommended for completion TME refused further surgery, or in the study by Al Najami et al., where only three out of 16 patients underwent completion surgery when recommended. Even if in the present literature the impact of completion surgery on short- and long-term outcomes is still debated [22,23], TME is still the gold standard treatment for these patients, in which early recurrence occurs when completion TME is not performed; in such cases, LE has the advantage of reducing the risk of clinical misdiagnosis of residual tumor [24].

On the other hand, in the included studies, in a WW program, the rate of rectum-preservation ranged between 85% and 95%, and the risk of failure of a rectal-sparing strategy was associated to local regrowth. Local regrowth typically occurred in the first two years of follow-up [6,25]. Most recently, data from the OPRA trial, a multicenter randomized phase II trial, including 225 WW patients following TNT, showed a local regrowth in approximately one third of the patients. Most interestingly, the DFS were similar in patients with TME at restaging and delay TME (salvage surgery) for local regrowth [7]. Moreover, in a large registry, approximately 25% of patients followed in a WW program had a local regrowth; anyhow, most of them (80%) had a salvage TME, resulting in an excellent 5-year disease-specific survival rate of 84.0% [6]. Despite these encouraging results, a meta-analysis comparing WW and TME previously reported a benefit in terms of DFS and local recurrence favoring TME [26]. However, the use of WW approach may be justified considering the low rate of patients non-candidable to a salvage TME after local regrowth (locoregional failure). In the MSKCC experience, approximately 20% of patients followed in a WW program experienced a local regrowth; however, all of these patients were treated with salvage surgery, and the pelvic control after salvage surgery was reached in 91% of cases, with an overall rectum-preservation rate of 82% [27]. In our meta-analysis, when comparing local disease and locoregional failure there were no differences between WW and LE. We decided to compare local regrowth and LR, since both are potential indications for rectal resection with TME, and may potentially impact on rectum-preservation. LE following nCRT also showed excellent local disease control, with a 5-year LR rate ranging between 7% and 10% [5,28]. In prospective studies approximately 60% of patients treated with LE for cCR or nCR were confirmed to have a pCR at final histopathological report, while 40% of the patients had a residual tumor ypT ≥ 1 [29]. In such cases, treating patients in a WW program may result in early local regrowth, which is potentially preventable using a LE approach. Another advantage of using LE is the possibility to extend a rectal preservation strategy to patients with nCR, which are not amenable to a WW program. Nevertheless, as also highlighted in Table 1, the definition of cCR and nCR are often very different according to the study protocol. Historically, the endoscopy-based definition of cCR proposed by Habr Gama et al. [12] included white area of the mucosa, with or without teleangiectasia, and scar, while any residual ulceration, also including a superficial ulcer and/or irregularity of the mucosa was defined as incomplete response. Along with the different clinical definition of response, a significant interobserver variation was reported in defining the clinical tumor response grade, and the accuracy in identifying cCR was reported to be approximately 80% [30]. In order to improve the evidences from rectal-sparing approaches studies, a recent consensus tried to uniform the definition and the outcomes in these studies, by defining the cCR as no residual palpable tumor (only residual small ulcer or scar), with no observable residual at MRI, and nCR as small smooth ulcer [31].

Delay in the diagnosis of tumor residual may result in an increased rate of DR. The rate of DR ranged between 0 and 33% in the WW group, and 6% and 18% in the LE group, and no difference was found at the meta-analysis. In large studies including WW patients, 8% of them were found to have a distant metastasis, and, when considering specifically patients with local regrowth, this rate increases up to 18–36% [6,27]. On the other hand, in the LE group, DR occurred in 13–20% of patients, with a 5-year distant-recurrence free survival was 90% [5,28]. However, there is an increasing interest in using TNT in patients with locally advanced rectal cancer, with the rationale of eradicating occult micrometastases and to prevent distant metastases. As a matter of fact, the meta-analysis showed that TNT was reported to be associated to an increased rate of pCR when compared to standard nCRT, and specifically to be associated to a decreased rate of DR, and an improved DFS [32,33]. In the OPRA trial DR occurred in approximately 25% and 27% of patients undergoing TME after TNT and salvage TME for local regrowth, respectively [7]; whereas Wang et al. reported an overall metastasis rate of 6%, and most of them in patients without a cCR or a nCR [19]. The risk of these rectal-sparing approaches is linked to the potential misdiagnosis of a non-major response which should be treated with a standard TME and may result in a locoregional or distant failure.

This study has several limitations, and the quality of evidence is very low as we reported using the GRADE approach. First of all, the number of included studies and patients is very low. Second, the included studies are mostly retrospective, and only one is prospective. Furthermore, the outcomes of our analysis were not considered as the main endpoint of the studies. This fact underlined the current absence of comparative studies directly comparing WW and LE for cCR or nCR following nCRT. Most of the current literature, compared WW or LE Vs TME, and high-quality evidence regarding specifically only rectal-sparing approaches in cCR, is lacking. In this setting the RESARCH trial, a multicenter observational study, was launched in 2017, and the preliminary results regarding patients treated with LE were published [29,34]. Even if comparative analysis according to the study protocol is not expected due to its observational design, the study will include both WW and LE. Third, we originally planned to also perform a survival meta-analysis. However, data regarding DFS or RFS of the included studies were widely heterogenous, and for this reason, a survival meta-analysis was not performed. Finally, as previously mentioned, there is a wide heterogeneity regarding the definitions (clinical response) and treatment, for example for nCRT (standard or TNT), the indications for completion surgery and the different study protocols, making the results of our meta-analysis hardly generalizable, but once again underlining the need of comparative studies including WW and LE.

## 5. Conclusions

Rectal-sparing approaches in patients with a cCR or nCR after nCRT are used in a selected clinical setting, and comparative studies comparing specifically WW and LE are still lacking. Our meta-analysis did not report differences between WW and LE in terms of rectum-preservation, local control, and distant metastases.

## Data Availability

The data presented in this study are available on request from the corresponding author.

## Supplementary Materials

The following supporting information can be downloaded at: https://www.mdpi.com/article/10.3390/cancers15020465/s1, Supplementary Table S1: GRADE Quality of Evidence.
