# Supplementary material for: Rectal Sparing Approaches after Neoadjuvant Treatment for Rectal Cancer: A Systematic Review and Meta-Analysis Comparing Local Excision and Watch and Wait
Source: Cancers (Basel). 2023 Jan 11;15(2):465. doi: 10.3390/cancers15020465 (PMC9856629; doi:10.3390/cancers15020465)
Supplement: Supplementary file 1 [file cancers-15-00465-s001.zip › cancers-2106322-supplementary.pdf]

Supplementary files
Table S1. GRADE Quality of Evidence

Certainty assessment							№ of patients		Effect		Certainty	Importance
№ of studies	Study design	Risk of bias	Inconsistency	Indirectness	Imprecision	Other considerations	Watch and Wait	Local Excision	Relative (95% CI)	Absolute (95% CI)		
**Overal rectal preservation rate**												
8	observational studies	very serious[a]	serious[b]	serious[c]	serious[d]	all plausible residual confounding would reduce the demonstrated effect	23/213 (10.8%)	29/188 (15.4%)	**OR 0.80** (0.31 to 2.01)	**27 fewer per 1.000** (from 101 fewer to 114 more)	⊕◯◯◯ Very low	CRITICAL
**Locoregional Failure**												
5	observational studies	very serious[a]	serious[b]	serious[c]	serious[e]	all plausible residual confounding would reduce the demonstrated effect	12/119 (10.1%)	12/133 (9.0%)	**OR 0.85** (0.20 to 3.66)	**12 fewer per 1.000** (from 71 fewer to 176 more)	⊕◯◯◯ Very low	CRITICAL
**Local Disease Free Survival**												
8	observational studies	very serious[a]	serious[b]	serious[c]	serious	all plausible residual confounding would reduce the demonstrated effect	46/213 (21.6%)	25/188 (13.3%)	**OR 1.60** (0.75 to 3.42)	**64 more per 1.000** (from 30 fewer to 211 more)	⊕◯◯◯ Very low	IMPORTANT
**Distant recurrence**												
8	observational studies	very serious[a]	serious[b]	serious[c]	serious	all plausible residual confounding would reduce the demonstrated effect	19/213 (8.9%)	23/188 (12.2%)	**OR 0.76** (0.37 to 1.55)	**27 fewer per 1.000** (from 73 fewer to 55 more)	⊕◯◯◯ Very low	CRITICAL

CI: confidence interval; **OR:** odds ratio

Explanations

a. Most of the studies are retrospective, and one phase II study. The rate of rectal preservation is often not included as main or secondary outcome. The definition of local recurrence and local regrowth is often overlapped or ambigous. Some studies included a wide study period. Different definition of inclusions criteria (complete or near-complete clinical response).
b. Most of the studies included complete and near-complete clinical response, however a indication for a treatment or the other are often not explained.
c. Some studies included comparison between Watch and wait, local excision, and total mesorectal excision, and the comparison between Watch and Wait and local excision were extracted from subgroup analysis,
d. Low number of events (rectal resection, or TME) due to patients refusal or unfit or not defined criteria for completion TME.
e. Locoregional failure often not defined by the Authors, and extracted from clinical and long-term outcomes.
